# In Vitro Digestive Analysis of Digestible and Resistant Starch Fractions, with Concurrent Glycemic Index Determination, in Whole Grain Wheat Products Minimally Processed for Reduced Glycaemic Impact

**DOI:** 10.3390/foods11131904

**Published:** 2022-06-27

**Authors:** John Monro, Suman Mishra

**Affiliations:** 1 The New Zealand Institute for Plant and Food Research Limited, Private Bag 11600, Palmerston North 4442, New Zealand; suman.mishra@plantandfood.co.nz; 2 Riddet Institute, University Avenue, Fitzherbert, Palmerston North 4474, New Zealand

**Keywords:** milling, cooking, starch fractions, glycaemic index, processing

## Abstract

Eight wheat products differing in texture (porridge vs. bread), grain fineness (fine, kibbled, intact), and cooking (raw vs. cooked), with pre-measured glycaemic indexes (GI), were analysed by in vitro amylolytic digestion to determine effects of processing to reduce GI on quantities of starch fractions differing in digestibility. The accuracy and precision of the in vitro analysis was assessed from its ability to concurrently predict clinical GI. In porridges, kernel intactness and lack of cooking reduced GI while increasing Type 1 (inaccessible) and Type 2 (ungelatinised) resistant starch. Porridge in vitro GI values (GI_iv_), calculated from the area under in vitro digestion curves minus estimated blood glucose disposal, were: raw fine, 26.3; raw kibbled, 12.6; cooked fine, 63.9; cooked kibbled, 44.1; and correlated closely with clinical GI values (R^2^ = 0.97). In bread, the negative association of kernel intactness and resistant starch with GI was seen in vitro but not in vivo. Bread GI_iv_ values were: roller milled flour, 67.4; stoneground flour 61.1; kibbled grain, 53.0; kibbled + intact kernel, 49.5; but correlation with clinical values was low (R^2^ = 0.47), and variability in the clinical results was high (clinical CV = 72.5%, in vitro CV = 3.7%). Low glycaemic potency of wheat by minimal processing was achieved by maintaining particle size, avoiding hydrothermal treatment, avoiding crushing and using a food matrix requiring little chewing for ingestion. Use of in vitro digestive analysis for high precision measurement of starch fractions with potential secondary health benefits was validated by accurate concurrent prediction of the glycaemic index but needed to account for effects of chewing.

## 1. Introduction

Two recent papers showed how different degrees of cooking and milling of wheat grain could be used to modify the glycaemic response in eight products [1,2]. Associated changes in starch digestibility were not measured. To fill this knowledge gap, we carried out timed in vitro digestive analysis of starch in the same products as were used in the foregoing clinical trials. The results are of interest for a number of reasons. In vitro analysis of starch digestibility in such a set of physiologically characterised samples may help to explain directly how the cooking and milling processes lead to the measured differences in glycaemic response. The analyses may also identify changes in starch fractions in wholegrains that have benefits, such as improved colonic health, secondary to the primary aim of reducing glycaemic potency. Having the two sets of data—from the clinical and in vitro digestive analyses—on the same samples also provides an opportunity to reassess the accuracy and relative precision of in vitro digestive analysis in developing products of low glycaemic impact. Furthermore, if data on starch digestion from timed sampling during in vitro digestion accurately predict a time-dependent physiological response, the starch analysis will have been physiologically validated.

Having timed the in vitro and in vivo responses for two texturally contrasting types of wholegrain products, porridges, and bread, also provides an opportunity to examine the effect of product type on glycaemic analysis. Porridges, such as mueslis, are grains ingested as slurries, require little chewing to swallow, and their starch component is derived wholly from partially intact grain fragments. In bread, on the other hand, grain particles are embedded in a relatively dry starch-based matrix which requires chewing and bolus formation to be easily swallowed. The inhibitory effect of coarse wheat grain structure on the glycaemic response to bread is largely eliminated during normal ingestion because of the effects of mastication [3]. Therefore, in vitro digestive analysis without an oral processing step that involves crushing should predict the glycaemic effects of grain particles consumed in porridges more accurately than when consumed in bread. Bread should be a less effective medium than porridges in which to capture the glycaemia-limiting benefits of grain structure retained by minimal processing.

A number of factors determine the effects of minimal processing on starch digestibility and have been exploited to make products of reduced glycaemic potency [4]. Native structures in wheat may inhibit starch digestion at a number of levels [5]. At the molecular level, the organisation of starch chains within granules retards digestion until disrupted by gelatinisation during hydrothermal processing [6,7]. At the morphological level, the starch density in the intact endosperm and an impenetrable seed coat may retard ingress of water for hydration and swelling of starch and limit access of digestive enzymes [8,9], particularly in wholegrain particles with an adherent seed coat. With such diverse factors acting to retard starch digestion, the interaction of particle size and cooking is likely to lead to a specific spectrum of nutritionally relevant starch fractions in any product. The starch complement may include rapidly digested (RDS), slowly digested (SDS), inaccessible (Type 1 resistant (RS1)), and ungelatinised (Type 2 resistant (RS2)) starches, with the proportions of the fractions depending on processing history [10,11]. Rapidly digested starch has been associated with postprandial hyperglycaemia [12], which may induce a subsequent transitional insulin-driven hypoglycaemic over-reaction in both healthy and metabolically impaired individuals [13]. More slowly digested starch induces a lower postprandial glucose peak but may sustain blood glucose above fasting into the late postprandial, inter-meal period [14]. Resistant starch fractions, which are, by definition, forms of dietary fibre, may act as prebiotics, with microbiota-mediated gut health benefits [15]. Such functional diversity in cereal starch fractions no doubt contributes to the multiple benefits recently linked to “carbohydrate quality” in whole grains [16]. It justifies analysing starch fractions in whole grain products because of the secondary health benefits they may have, even if developed primarily for low glycaemic impact.

Establishing a valid association between changes in starch fractions and glycaemic impact would require measurement of starch digestion in the same material as was responsible for the glycaemic response, with a sampling of intestinal contents. However, if an in vitro digestion method used to determine starch fractions releases digestion product at a rate that correlates closely with the glycaemic response, it is likely to provide an accurate estimate of the starch fractions in vivo associated with the response and would validate the method. A method based on clinical data has been developed. It provides simulated blood glucose response curves by progressively subtracting estimated blood glucose disposal from available carbohydrates released in the course of in vitro digestion [17]. The area under these curves may be used to obtain an accurate in vitro estimation of glycaemic index (GI) by the same trapezoid summation analysis as is used in the clinical determination of GI [18]. If the GI values obtained during in vitro starch analysis accurately predict clinical GI values determined on the same material, it would provide concurrent validation of the method.

An aim of the present research was to determine how the content of nutritionally relevant starch fractions in wheat grain products would change as a result of processing to reduce the glycaemic potency of the products. A second aim was to test the correlation between GIs of the products predicted from in vitro digestion and GIs determined from the incremental area under the postprandial blood glucose response curves, as a test of the accuracy of the in vitro digestive analysis, and as a concurrent validation of the starch, fractions measured. A further aim was to determine whether the type of product in which kibbled wheat structure was ingested affected the survival of the low glycaemic and resistant starch properties of the kibbled grains. Two auxiliary experiments were conducted on the effects of cutting, crushing and cooking, respectively, on the digestibility of starch in cereal grains to demonstrate the effect of the individual physical processes on the glycaemic potency of cereal products. Finally, the research illustrates how processing steps taken to reduce the glycaemic impact of cereal products may lead to secondary changes in starch fractions considered to be of potential benefit to gut health [15].

## 2. Materials and Methods

### 2.1. Samples

Four bread and four porridge samples (Table 1) were prepared for clinical trials, and sub-samples supplied for the in vitro analyses reported here by the Dept. of Food Science, University of Otago. Preparation procedures have been detailed in publications presenting the clinical results [1,2]. The bread were nutrient matched, as were the porridges. A commercial white bread (Tip Top White) purchased at a local supermarket was included as an external reference, representing the white bread standard for which a GI of 70 is assumed [19]. The particle size of the flours was <150 μm, while the kibbled grains were >1680 μm [1,2]. The four experimental bread were baked using the same standard commercial procedures.

The cooked porridges were made by heating the grain preparations in water at 85 °C for 15 min and served at 65 °C to prevent starch retrogradation. The porridges were served with yoghurt in portions required to deliver a 50 g carbohydrate dose ^1^.

### 2.2. In Vitro Digestion

The in vitro digestion has been described in detail elsewhere [18]. Briefly, the bread was coarsely fragmented by rubbing gently through a 0.5 cm grating with care taken not to crush any particles. The porridge samples were dispersed gently in the digestion medium with a spatula, also with care not to crush particles. The samples were then subjected to an in vitro digestive analysis, which included a 30 min gastric phase followed by intestinal digestion lasting 150 min. During the intestinal phase, samples of the digestion medium were removed from the digestion pots for analysis of the accumulation of carbohydrate digestion products with time.

The digestions were carried out in duplicate in 70 mL specimen pots (LabServ, LBS 30002, Thermo Fisher Scientific, Auckland, New Zealand) placed in a custom-built heating block on a 15-place magnetic stirrer. A sub-sample (2.5 ± 0.01 g) of each bread was accurately weighed into each pot, 30 mL of water added, followed by 1.0 mL of 1 M HCl and 1 mL of 10% pepsin protease (Sigma-Aldrich, P-7125, Merck Life Science, Auckland, New Zealand) dissolved in 0.05 M HCl. The samples were incubated at pH 2.5 and 37 °C for 30 min to simulate gastric digestion.

The intestinal phase was initiated by adjusting the samples to pH 6.3 by adding 2 mL of 1 M NaHCO_3_ and 5 mL of 0.2 M Na maleate buffer pH 6.3 and made to the full (53 mL) mark with distilled water. Intestinal digestion, during which carbohydrate digestion was monitored, was initiated by adding 0.1 mL of amyloglucosidase (Megazyme, E-AMGDF, Megazyme, Wicklow, Ireland) and 1.0 mL of 1% pancreatin (Sigma-Aldrich, P-7545, 4 × USP, Merck Life Science, Auckland, New Zealand) solution in maleate buffer to the digestion pots, which were stirred at 130 rpm and 37 °C. Samples (0.5 mL) were removed to ethanol (2.0 mL) at 0, 10, 20, 40, 60 and 120 min and immediately mixed to stop digestion and precipitate any undigested starch. At 120 min, the digests were homogenised (Omni-GLH with an S18N-19G dispersing tool) to a slurry in the digestion pots, a further 0.1 mL of amyloglucosidase added, and the samples digested for a further 30 min to capture any starch protected by food structure before a final 0.5 mL sample was removed to ethanol.

The ethanolic samples were centrifuged and a sub-sample of the supernatant given a secondary digestion, with 1% amyloglucosidase and 1% invertase, to simulate brush border processing and convert all 80% ethanol-soluble fragments from digestion of starch (short dextrins, maltose) to glucose for analysis. Soluble sugars were then measured as grams of glucose equivalents (GE) using the standard dinitrosalicylic acid colourimetric procedure [20].

Starch fractions determined in the above analysis were:

Rapidly digested starch (RDS)—starch digested in vitro up to 20 min. RDS refers solely to the time period of digestion, although it will contain glucose from starch species that differ in their intrinsic rates of digestion.

Slowly digested starch (SDS)—starch digested between 20 and 120 min in vitro.

Resistant starch Type 1(RS1)—starch that is not digested because amylase access is restricted by food structure. It was measured as the increase in sugar release at 150 min when the sample had been given secondary digestion after homogenising at 120 min.

Resistant starch Type 2 (RS2)—starch resistant to digestion as a result of native molecular structure. Hydrothermal processing converts Type 2 RS to digestible gelatinised starch as long as the starch is able to hydrate. RS2 was measured as difference between total starch in the fully cooked dispersed as the difference between total starch (TS) digested in the dispersed fully cooked sample and the sum of the preceding fractions (RS2 = TS − (RDS + SDS + RS1)).

### 2.3. In Vitro GI Values

**Figure 1:**
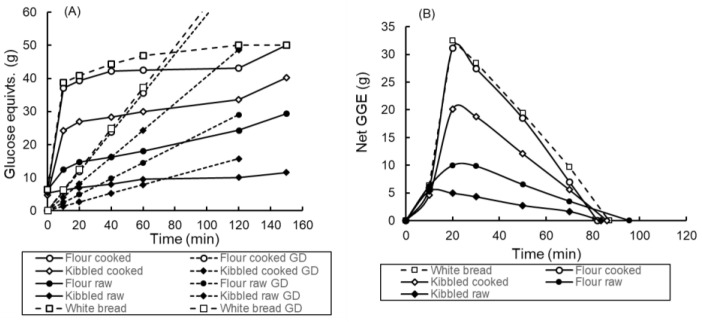
(**A**) Digestogram for porridge samples (all 50 g potentially available carbohydrate) differing in particle intactness and cooking. Samples were homogenised at 120 min and digestion continued to 150 min. Points are means of duplicate samples. Mean absolute deviation from mean = 1.2 g. Glucose disposal (GD) baselines corresponding to each digestion curve are shown. (**B**) Net glycaemic glucose equivalents were determined from the difference between each digestion curve and its corresponding GD baseline in Figure 1A. Potential available carbohydrate content was assumed to be that measured in the cooked homogenised flour sample at 150 min digestion.

**Figure 2:**
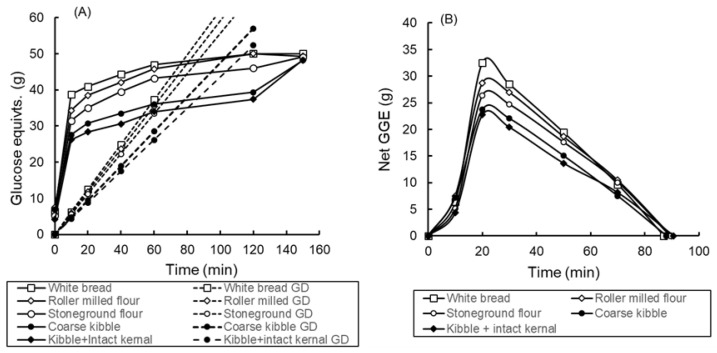
(**A**) Digestogram for bread samples (all 50 g available carbohydrate) containing wheat preparations differing in degree of milling. Samples were homogenised at 120 min and digestion continued to 150 min. Points are means of duplicate samples. Mean absolute deviation from mean = 1.2 g. Glucose disposal baselines (GD) for each curve are shown. (**B**) Net glycaemic glucose equivalents (GGE) were determined from the difference between each digestion curve and its corresponding GD baseline in Figure 2A. Potential available carbohydrate content in the wholegrain samples was assumed to be that measured in the homogenised, roller milled sample at 150 min.

In vitro glycaemic index (GI_iv_) was determined from the digestion curves as previously described [21]. Curves of cumulative sugar release from the products were determined, as well as theoretical cumulative glucose disposal over the same time (Figure 1 and Figure 2). Equations for glucose disposal (GD) rate as a function of glycemic glucose equivalent (GGE) intakes had previously been determined in clinical studies [18] and were applied to in vitro values adjusted to serving size to provide realistic GD lines specific for each of the individual digestion curves. The difference (net GGE) between the lines of GGE release and GD provided a simulated blood glucose response from which the area under the curve (AUC) was determined by trapezoid summation. Comparison of the AUC for a product with the AUC for white bread with an assumed GI of 70 was used to derive the GI_iv_ of the product:

GI_iv_ = (AUC_product_/AUC_white bread_) × 70

### 2.4. Clinical GI Values

The clinical GI values were determined from the mean iAUC values given in the publications describing blood glucose responses to the porridges [1] and bread [2], subsequently subjected to the in vitro digestive analysis described in the present paper.

### 2.5. Confirmation of Cutting, Crushing and Cooking Effects

Two additional experiments were conducted to show the role that three factors may play in the observed differences between treatments in the main study of porridge and bread: (A) The role of cutting and crushing in digestibility of cooked wheat grain; (B) The role of cooking in digestibility of crushed grain, using wholegrain rolled oats.

In Experiment A, wheat grains were allowed to hydrate overnight and cooked for 10 min in a glass tube in a boiling water bath. The cooked grains were digested individually either intact, sliced equatorially with a sharp razor blade, or crushed to 1 mm between glass sides, with 5 grains for each treatment. A scaled-down version of the in vitro digestion (above) was used with sugar release measured at 20 min (RDS), 120 min (RDS + SDS) and after homogenising with further digestion (RDS + SDS + RS1).

In Experiment B commercially available wholegrain rolled oats (Harraway’s, Dunedin, New Zealand) were digested using the full-scale in vitro digestion procedure (above; 2.5 g/50 mL) either as is or after cooking for 10 min in a boiling water bath for determination of RDS, RDS + SDS, and after homogenising, total starch.

### 2.6. Data Analysis

Mean values were calculated for all time points in the digestion. Precision of the analyses was estimated as the mean absolute deviation between duplicate values for sugar release per 100 g sample.

Values for total potentially available carbohydrates in the products were obtained from the 150 min value for the homogenised cooked version of the product (bread or porridge) made from the finest particle size flour. This would correspond to a standard available carbohydrate analysis.

Standard deviations (SD) of the clinical GI values (SD_GI_) were calculated from the given SDs of the IAUC values used to calculate GI using the formula:

SD_GI_ = SD (X × 100/Y) = ((Mean x/Mean y) × sqrt((SD x/Mean x)^2^ + (SD y/Mean y)^2^)) × 100

where X and Y are the IAUCs of the sample and glucose reference, respectively [1,2].

## 3. Results

The analysis of soluble sugar release during digestion of all samples was conducted in a single batch, in duplicate, with good precision; the average between-duplicate deviation was 1.2 g/100 g sample (Table 2). For the Tip Top Supersoft white bread reference sample, the total available carbohydrate measured as total sugar released by 150 min of pancreatic/amyloglucosidase action was 44.7 g/100 g sample. This compared with a value of 48.2 g/100 g in the nutrient information panel (which is usually determined by difference so slightly overestimates true available carbohydrate) of the reference bread, indicating that the digestive analysis used in the present study was reasonably accurate.

### 3.1. Porridges 

The initial rate and extent of starch digestion, as indicated by RDS, were affected by processing more in the porridges than in the bread, ranging from 3.5 to 19.9 g/100 g sample in the porridges compared with 21.4 to 29.0 for the bread. (Table 2). Rapidly digested starch (RDS) as a percentage of total starch was least in the uncooked porridge samples (raw flour 29.4%; raw kibbled 14%) (Table 2) and increased considerably upon cooking (cooked flour, 78.6%; cooked kibbled, 53.8%). RS2 was a large proportion of total starch in the uncooked porridge samples (raw flour, 41.2%; raw kibble, 76.7%). Approximately 80% of the raw kibbled sample was dietary fibre in the form of resistant starch, compared with about 14% in the cooked wholemeal flour.

### 3.2. Bread 

Across all bread, the rate of starch digestion and the distribution of starch fractions differed (Table 2). The most rapidly digested starch was in the bread based on flour, which also contained the least resistant starch. The fine kibble (stoneground) contained less RDS than the flour bread and more SDS than the other bread, but still a relatively low content of RS1. In the bread containing coarse kibble, and coarse kibble/whole wheat, the RDS was lowest, and there was a notable increase in RS1. In the coarse kibble/whole wheat bread, a small proportion (12.6% of total starch) of RS2 appeared, so about 30% of the starch (RS1 + RS2) was in the form of dietary fibre (Table 2).

### 3.3. Glycaemic Indexes

**Figure 3:**
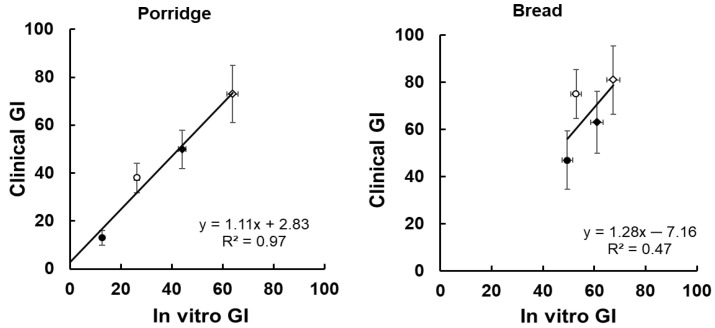
Correlations between GI values determined in vitro and in vivo [1,2], for porridges and bread. For clinical values (y axis) means ± SEMs are shown. For in vitro values (x-axis) error bars are analytical SDs based on average CVs of 3.5% (porridge) and 3.8% (bread). Porridges: fine cooked (◊), coarse cooked (♦), fine raw (○), coarse raw (●). Bread: roller milled flour (◊), stoneground flour (♦), kibbled grain/stone ground flour (○), intact grain/kibbled grain/stoneground flour (●).

The digestograms for the bread, which were all cooked, were much more tightly grouped than those of the porridges (Figure 1 and Figure 2). The uncooked porridges gave much lower in vitro and clinical GI values than the cooked samples (Table 3). The in vitro GI values accurately predicted clinical values for the porridges (R^2^ = 0.97; y = 1.11x = 2.83) but not for the bread (R^2^ = 0.47, y = 1.28x − 7.16) (Figure 3). For all products combined the correlation was R^2^ = 0.88, y = 1.11x + 2.31.

The in vitro GI values were determined with much greater precision than the clinical values because they were unaffected by individual differences in glycaemic responsiveness.

RDS as a percentage of TS (x) closely predicted clinical GI (y) for porridges (R^2^ = 0.99, y = 1.03x − 2.05), but less closely for the bread (R^2^ = 0.68, y = 0.97x − 2.37). Across all products the percentage of starch that was RDS was a strong predictor of both the clinical and in vitro GI values (GI_clin_: R^2^ = 0.92, y = 0.95x + 3.21; GI_iv_: R^2^ = 0.97, y = 0.83x + 2.25, where y = GI and x = RDS as a percentage of TS).

### 3.4. Supplementary Experiments

**Figure 4:**
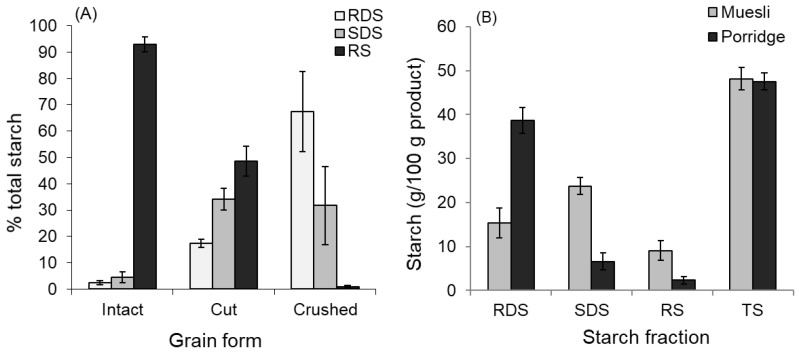
Effects of physical state and cooking on starch fractions in cereals: (**A**) Effect of cutting versus crushing (chewing) of cooked wheat kernels on starch digestion. (**B**) Effect of complete cooking (porridge) versus no cooking (muesli) of a crushed partially cooked (steamed) cereal product (rolled oats). Means ± SD. (RDS = rapidly, SDS = slowly, RS = resistant and TS = total starch).

In the intact cooked wheat grain, starch was almost completely protected from amylase digestion. However, when the grain was crushed, the starch was almost completely digested (Figure 4A). When the grain was cut without crushing, about half of the RS was digested.

The results with the rolled oats show that crushing grain alone will not render starch digestible if it is not fully cooked. Rolled oats are a partially cooked (steamed) grain that has been crushed during rolling. In the form of muesli, its starch was only slowly digested, but upon cooking as hot porridge, there was a large increase with in vitro digestible starch fractions (Figure 4B), with RDS increasing as a proportion of total starch from 32 to 81%, and GI_iv_ based on the area under the digestion curve increasing from 51.3 to 83.2.

## 4. Discussions

The results have shown the extent to which increased particle size and reduced gelatinisation, which may be part of minimal processing of whole grains for reduced glycaemic potency, lead to changes in starch fractions that could have secondary health benefits. Amongst these changes, an increase in RS may make an important contribution to gut health through its prebiotic effects in the colon, with numerous secondary health benefits [15]. Processes increasing RS in bread could, in theory, substantially improve dietary fibre intakes and associated gut health. In the bread containing 30% intact and 30% kibbled kernels, for instance, about 30% of the starch was in the form of resistant starch. Two average (45 g) slices of the bread, each containing 16 g of starch, would provide about 10 g of dietary fibre as RS, probably combined with 3–4 g of non-starch polysaccharide, thus potentially providing almost half of the daily recommended intake of about 30 g dietary fibre. However, the measured proportion of RS will be the maximum possible because a proportion of the RS1 may be lost through structural disintegration by chewing during normal ingestion. Thus, the in vivo contribution from intact grains may be lower in reality than was suggested by the in vitro results and would depend on the chewing characteristics of the consumer [22]. Nevertheless, in the present study, the reduction in GI of the intact kernel bread of about 20 GI units compared with the wholemeal bread was consistent with the in vitro reduction in RDS and increased in RS to about 30% of total starch.

A typical serving of porridge or muesli containing 50 g of wholegrain, with a “carbohydrate” (total starch) content of about 35 g would, according to Table 2, provide dietary fibre in the form of resistant starch ranging from 4.9 g for fine cooked, to 17.8 g for fine uncooked, to 28 g for kibbled uncooked, and 11.5 g for kibbled cooked. Thus, the uncooked kibbled grain could, in theory, provide most of the recommended daily intake of dietary fibre to augment the soluble and insoluble non-starch polysaccharide already present. Thus, the grain would be a valuable source of mixed dietary fibre in cereal mixtures consumed as intact and uncooked or partially uncooked grains, such as muesli. However, as the results in Figure 4 showed, dietary fibre in the form of resistant starch is extremely susceptible to conversion to available carbohydrates by cooking and crushing treatments, which provides the opportunity to develop products with specific physiological effects by careful control of processing conditions.

An important advantage of consuming resistant starch in the form of intact and partially intact cereal kernels in whole grain products is that it is accompanied by bran. While RS may improve gut health by acting as a prebiotic substrate for the microbiota [15], it adds little to faecal bulk because it is fermented. In contrast, the bran is a protective integument that resists bacterial attack, so it survives colonic transit and contributes more effectively to faecal bulk [23]. Faecal bulking is an extremely important complementary role of dietary fibre in maintaining gut function and health, with many downstream benefits [24]. Cereal products with the bran fraction removed are generally inadequate as sources of distal colonic bulk, while those that retain the bran have a high faecal bulking capacity [25].

The two supplementary experiments on cooked wheat kernels (Figure 4A) and rolled oats (Figure 4B) respectively illustrated the importance of several factors determining the glycaemic potency of wholegrain cereals, namely cutting and crushing of intact cooked grains as would occur in kibbling and chewing to form a bolus, and starch gelatinisation as a result of hydrothermal processing. Both crushing cooked grain and cooking crushed grain largely eliminated the protective effect of structure at the plant morphological and starch granule levels. Cooking and crushing effects on glycaemic impact will combine when solid cooked wholegrain products, such as bakery products containing coarse kibbled and intact kernel particles in a dryish food matrix, are ingested because chewing will be induced as an essential part of bolus formation for swallowing. However, in products such as muesli, in which the starch is incompletely gelatinised, and the product is swallowed in the form of a slurry rather than a bolus, the combined effects of incomplete starch gelatinisation and grain structure may substantially modify the glycaemic index, as seen in the wide spread of GI values for porridge (Figure 3). In cooked porridge, the effect of grain structure is likely to escape the effects of chewing, as the grains are swallowed in a slurry lubricated by gelatinised starch.

The high correlation between in vitro and in vivo GI values for the porridges suggests that in vitro digestion gives a true indication of the functional states of starch in vivo because the starch fractions and the in vitro GI values were based on the same starch digestion data. Furthermore, the physical form in vivo and in vitro would have been similar because bolus formation was not required for ingestion. The reason for the weaker correlation between the in vitro and in vivo GI values in the bread is probably because the population density of wholegrains was reduced by the flour-based bread matrix, The “hydrothermal” process of baking would have converted most of the RS1 to RDS, and the data were subject to large errors intrinsic to clinical GI determinations [26]. The clinical study was probably underpowered to measure the differences between the bread in glycaemic impact. The figure illustrates the value of the precision of in vitro analysis of glycaemic impact compared with the unavoidable imprecision of in vivo measurements due to large individual differences. Although the intact grains were still able to yield a lower GI than the wholemeal, the difference was due partly due to Type 2 (ungelatinised) RS (Table 3), suggesting that the intact grain structure had inhibited endosperm hydration and/or starch gelatinisation.

In an earlier study of bread containing fully hydrated, cooked, coarse grain, swallowing the bread without chewing lowered glycaemic impact, but the effect was largely eliminated when the bread was ingested normally [3]. Presumably, chewing during bolus formation [27], prolonged exposure to amylase during bolus persistence in the stomach, and other physical and enzymatic processes that the stomach uses to disintegrate foods obliterated the glycaemia-inhibiting effects of grain structure [28,29].

The results of the present study indicate that if coarsely kibbled grains are used to lower the glycaemic potency of foods, the foods containing them should be moist enough or textured to avert the need and urge to form a well-chewed bolus containing crushed particles before swallowing. Secondly, as RS2 formed such a substantial portion of the products with the lowest in vitro and clinical GIs (Figure 4, Table 2), limited hydrothermal processing should be part of minimal processing for low glycaemic impact wholegrain foods. Thus, a muesli-type product would be preferable to hot porridge for glycaemic control.

The present study has also shown how in vitro digestive analysis can be a useful tool in developing whole grain products for low glycaemic potency and/or improved prebiotic potential based on restricting the conversion of RS to digestible starch. The correlation between in vitro GI and clinical GI across all eight products was R^2^ = 0.88, consistent with the value of R^2^ = 0.90 obtained with 24 carbohydrate foods of diverse types [21], and with earlier research showing that RDS predicts glycaemic response [12]. The present research has confirmed that RDS is an accurate predictor of glycaemic response, at least in simple cereal products. A great advantage of the in vitro GI analysis over clinical GI is its far greater precision (Table 3, Figure 3), with CVs of about 5%, typical of laboratory analysis. Although clinical determination is mandatory for values to be assigned to consumer products, during the development phase, in vitro analyses can provide economic and precise identification of the best products to be taken into far more costly clinical trials. They can therefore allow greater speed, economy and accuracy than going directly from formulation to clinical trials in product development for glycaemic control.

However, as the present study has shown, if in vitro digestion is to be used to gauge the relative glycaemic impact of whole grain foods, such as experimental preclinical cereal products, it is important that the food be disintegrated in a way that accurately replicates the physical effects of ingestion, such as crushing (Figure 4). For foods that do not need to be chewed before swallowing, such as a porridge or muesli slurry, the in vitro GI estimation proved to be reasonably accurate in this study.

If the in vitro procedure is to be applied beyond simple wholegrain cereal products, to produce low glycaemic impact composite products, the influence of food components that affect gastric emptying rate may need to be considered. Components such as fat, highly viscous polysaccharides, and organic acids may need to be made constant across comparisons so that effects of cereal and starch structure are not confounded by separate effects of food components on digestion and glucose absorption. Once the role of cereal structure has been defined under controlled conditions, other factors may be included to determine their additional contribution to an overall improvement in the nutritional attributes of a product.

This paper, by showing the substantial impact of factors, such as particle size, pre-hydration, cutting, crushing, and gelatinisation on the glycaemic potency of wholegrains, has highlighted a set of variables that can be systematically varied during minimal processing of grains. The products generated could create a matrix of minimally processed wholegrains for use in numerous composite products of predictable glycaemic impact.

The present study suffered from some limitations imposed by the fact that the primary aim of the study was to measure the effects of milling and cooking of wheat on human glycaemic responses to wheat products, with a necessarily limited number of samples. The design of the clinical study meant that changes in starch fractions and glycaemic responses as a detailed function of isolated processing variables could not be determined. However, the availability of the clinical data and the products responsible provided an excellent opportunity to retest in vitro digestive analysis and obtain some explanation for the clinical effects in terms of the effects of processing on starch fractions.

## 5. Conclusions

Minimal processing to preserve structure in kernel fragments and whole grains, and molecular organisation in starch granules, may moderate the rate of digestion of starch in cereal products, leading to low glycaemic potency and an increase in resistant starch fractions. A dual benefit of improved glycaemic control and improved gut health may therefore be obtained. In vitro digestive analysis can be an accurate and economical tool in the preclinical development of healthier wholegrain products, provided the effects of in vivo ingestion on food structure are allowed.

## Figures and Tables

**Table 1:** Samples of wholegrain wheat products supplied for in vitro glycaemic impact analysis ^1^.

Sample	Product Type	Cooking	Whole Grain Components	Proportions (%)
1	Porridge	Uncooked	Fine ^2^	100
2	Porridge	Cooked	Fine	100
3	Porridge	Uncooked	Kibbled ^3^	100
4	Porridge	Cooked	Kibbled	100
5	Bread	Cooked	Flour, roller milled (RM)	100
6	Bread	Cooked	Flour, stoneground	100
7	Bread	Cooked	Roller milled (RM) flour + coarse kibble	50:50
8	Bread	Cooked	RM flour + coarse kibble + intact kernel	40:30:30

^1^ Based on published data [1,2]. ^2^ Fine ≤ 150 µM. ^3^ Kibbled ≥ 1680 µM.

**Table 2:** Content of starch fractions in wholegrain wheat products (g/100 g sample) ^1^ and percent contribution to total starch (TS) in each sample (%TS).

		RDS		SDS		RS1		RS2		TS
	Sample and Components	Mean (% TS)		Mean (% TS)		Mean (% TS)		Mean (% TS)		Mean
	*Porridge*									
1	Fine, raw	7.43 (29)		4.87 (19)		2.57 (10)		10.4 (41)		25.3
2	Fine, cooked	19.9 (79)		1.96 (7.8)		3.46 (14)		0 (0.0)		25.3
3	Kibbled, raw	3.50 (14)		1.58 (6.3)		0.74 (3.0)		19.1 (77)		24.9
4	Kibbled, cooked	13.6 (54)		3.35 (13)		3.43 (14)		4.92 (19)		25.3
	*Bread*									
5	Flour, roller milled (RM)	29.0 (78)		7.0 (19)		1.21 (3.2)		0 (0.0)		37.2
6	Flour, stoneground	25.4 (69)		9.3 (25)		2.33 (6.3)		0 (0.0)		37.0
7	Flour, RM + kibbled	23.1 (64)		6.5 (18)		6.68 (18)		0 (0.0)		36.3
8	Flour, RM + kibbled + intact	21.4 (51)		6.8 (16)		8.16 (20)		5.23 (13)		41.6
	White bread (reference)	36.5 (82)		8.2 (18)		0 (0.0)		0 (0.0)		44.7

^1^ Means of duplicate determinations. (Mean of absolute deviations from mean = 1.22 g, SD = 1.04 g).

**Table 3:** GI values determined from incremental areas under the net GGE curves from in vitro digestion (Figure 1 and Figure 2) compared with GI values calculated from published areas under the curves of blood glucose response to the same products.

		GI In Vitro		Clinical GI ^1^		
Sample and Components		Mean	±SD ^2^	Mean	±SD ^1^	±SEM
	*Porridge*					
1	Fine, raw	26.3	±0.9	38	±26	±6.1
2	Fine, cooked	63.9	±2.2	73	±51	±12.0
3	Kibbled, raw	12.6	±0.4	13	±13	±3.1
4	Kibbled, cooked	44.1	±1.5	50	±34	±8.0
	*Bread*					
5	Flour, roller milled (RM)	67.4	±2.6	81	±56	±14.5
6	Flour, stoneground	61.1	±2.4	63	±51	±13.2
7	Flour, RM + kibbled	53.0	±2.1	75	±40	±10.3
8	Flour, RM + kibbled + intact	49.5	±2.0	47	±48	±12.4

^1^ Values calculated from published mean iAUC values and associated standard deviations [1,2].^2^ Based on mean coefficient of variation (CV) of carbohydrate release at each time point during in vitro digestion: Bread, CV = 3.8%; Porridge CV = 3.5%.

## Data Availability

The data presented in this study are available on request from the corresponding author.
